# Novel 3D µtissues Mimicking the Fibrotic Stroma in Pancreatic Cancer to Study Cellular Interactions and Stroma-Modulating Therapeutics

**DOI:** 10.3390/cancers13195006

**Published:** 2021-10-06

**Authors:** Kunal P. Pednekar, Marcel A. Heinrich, Joop van Baarlen, Jai Prakash

**Affiliations:** 1 Engineered Therapeutics Group, Department of Biomaterials Science and Technology, Technical Medical Centre, University of Twente, 7500 AE Enschede, The Netherlands; k.p.pednekar@utwente.nl (K.P.P.); m.a.heinrich@utwente.nl (M.A.H.); 2 Laboratorium Pathologie Oost-Nederland (LabPON), 7555 BB Hengelo, The Netherlands; j.vanbaarlen@labpon.nl

**Keywords:** pancreatic ductal adenocarcinoma, 3D in-vitro model, 3D µtissues, primary pancreatic stellate cells, cancer-associated fibroblasts, tumor microenvironment, cell contraction, collagen hydrogel

## Abstract

**Simple Summary:**

Pancreatic ductal adenocarcinoma (PDAC) is the most prevalent and aggressive type of pancreatic cancer with a low 5-year survival rate of only 8%. The cellular arrangement plays a crucial role in PDAC, which is characterized by a highly fibrotic environment around the tumor cells, preventing treatments from reaching their target. For the development of novel drug candidates, it is crucial to mimic this cellular arrangement in a laboratory environment. We successfully developed a reproducible three-dimensional cell culture model that demonstrates the PDAC characteristic arrangement and showed a PDAC relevant gene profile when comparing with the genetic profile of PDAC patients. We finally demonstrated the use of the model for the evaluation of novel anti-fibrotic therapy against PDAC by studying drug-induced reduction of fibrosis in PDAC enabling nanoparticles to penetrate and reach the tumor cells. This model is useful for the evaluation of novel treatments against PDAC in a biologically relevant manner.

**Abstract:**

Pancreatic ductal adenocarcinoma (PDAC) is a highly aggressive tumor type with low patient survival due to the low efficacy of current treatment options. Cancer-associated fibroblasts (CAFs) in the tumor microenvironment (TME) create a dense fibrotic environment around the tumor cells, preventing therapies from reaching their target. Novel 3D in vitro models are needed that mimic this fibrotic barrier for the development of therapies in a biologically relevant environment. Here, novel PDAC microtissues (µtissues) consisting of pancreatic cancer cell core surrounded by a CAF-laden collagen gel are presented, that is based on the cells own contractility to form a hard-to-penetrate barrier. The contraction of CAFs is demonstrated facilitating the embedding of tumor cells in the center of the µtissue as observed in patients. The µtissues displayed a PDAC-relevant gene expression by comparing their gene profile with transcriptomic patient data. Furthermore, the CAF-dependent proliferation of cancer cells is presented, as well as the suitability of the µtissues to serve as a platform for the screening of CAF-modulating therapies in combination with other (nano)therapies. It is envisioned that these PDAC µtissues can serve as a high-throughput platform for studying cellular interactions in PDAC and for evaluating different treatment strategies in the future.

## 1. Introduction

Pancreatic ductal adenocarcinoma (PDAC) accounts for up to 80% of all pancreatic cancers and is characterized by high aggressiveness, late diagnosis and challenging surgical resection due to its complicated localization, as well as a high resistance to treatments [1,2,3]. In particular, the low efficacy of current treatments such as chemotherapy contribute to the low five-year patient survival of only 8%, which, even with intensive treatment, might only be prolonged by a couple of months. Despite tremendous effort in the development of new therapeutics in the recent years, the overall mortality and incidence continuously increased over the last years and is predicted to further increase in the future [3,4].

In particular, the increasing number of PDAC patients demonstrates the urgent need for novel therapeutics; however, only a fraction of potential therapeutics successfully reaches the clinics. One of the major reasons for this low success rate is the tumor microenvironment (TME) in PDAC, in which the tumor stroma forms a dense and hard to overcome barrier for novel therapeutics [1,5,6,7,8,9]. In PDAC this barrier is characterized by an abundance of cancer-associated fibroblasts (CAFs), largely originating from pancreatic stellate cells (PSCs), which produce large amounts of extracellular matrix (ECM) proteins such as collagen, fibronectin or hyaluronic acid, creating a dense and desmoplastic environment [5,7,10,11].

The increasing understanding of the PDAC TME and its limiting effects on drug penetration and efficacy, also demonstrated that conventional 2D monolayer cultures of cells are insufficient to mimic this complex environment, as cancer cells and CAFs can only exhibit their natural behavior in a biologically relevant 3D environment [1,12]. Animal models arguably offer such a 3D environment and have been widely applied in recent years to assess the efficacy of novel therapeutics; however, these models often lack the human species (e.g. syngeneic models) or present a mixture of mouse and human species (e.g., xenograft model) and often display structural difference of cancer cells and the TME around them [13,14]. To better mimic the human PDAC in a pre-clinical environment, 3D in-vitro models have been developed in recent years, allowing us to evaluate novel therapeutics and study cellular interactions in humans in a controlled and cost-effective way before embarking on potential animal studies [1,12]. Particularly in PDAC, mimicking the dense stroma in a controlled 3D in-vitro model might facilitate the faster development of novel therapeutics as candidates can be screened before reaching more time- and cost-intense clinical stages.

We have recently developed a 3D in-vitro model including cancer cells and PSCs in the form of PDAC heterospheroids and successfully demonstrated their use for the evaluation of novel drug candidates such as small bioactive lipids, peptides or nanomedicine formulations [15,16,17,18,19]. Recently, organoids have gained increased attention among researchers due to their capability to mimic tumorigenic stages of PDAC in a controlled environment, which eventually led to the identification of different subtypes of CAFs in PDAC [8,20,21]. While spheroids and organoids demonstrate promising application to study cellular interactions in PDAC, as well as serve as a platform for drug evaluation, a major disadvantage of these systems is the lack of control on the cellular arrangements similar to in vivo. These models include the cellular composition of PDAC; however, they lack the spatial arrangement and therefore do not represent the dense barrier TME forming in vivo. Recently, 3D bioprinting has demonstrated its capability to generate 3D in-vitro models of PDAC, including cancer cells and PSCs, based on additive manufacturing that replicate the spatial arrangement of cells in a more biologically relevant manner [22,23]. However, 3D bioprinting is still a comparably complex fabrication technique, involving special equipment (e.g., a bioprinter) and training, and is highly time-consuming, not achieving the high throughput that is required to develop novel therapeutics.

In this study, we demonstrate the generation of novel PDAC microtissues (µtissues) based on the combination of Panc-1 cancer cell spheroids embedded in a collagen hydrogel including patient-derived primary PSCs. We generated µtissues with Panc-1 core surrounded by a dense CAF-rich environment. We further demonstrate the expression of PDAC relevant genes in our novel 3D µtissues by examining their genetic profile and compare the profile with publicly available transcriptomic data from PDAC patients, displaying a high similarity between our µtissues and PDAC patients. Furthermore, we show that PSCs do not only generate a strong barrier around the Panc-1 core but also drastically increase Panc-1 cancer cells proliferation. Finally, we present the capability of our µtissues to be used as a tool for the evaluation of novel drug candidates by using a peptide, AV3, that has been recently developed in our group, to inhibit PSCs contraction in combination with silica nanoparticles representing a combination treatment [19]. The proposed 3D in-vitro model is likely to advance the field of 3D in-vitro models of PDAC by offering a simple and reproducible platform that mimics the cellular composition and arrangement, as well as crucial characteristics of PDAC in a biologically relevant fashion.

## 2. Materials and Methods

### 2.1. Immunofluorescent Staining on Human PDAC Sections

Anonymous paraffin-embedded human PDAC sections were obtained from Laboratory Pathology East Netherlands (LabPON; Hengelo, The Netherlands). Sections were deparaffinized in xylene and rehydrated in a serious of ethanol followed by MilliQ water. Antigen retrieval was performed by heat induction at 95°C in citrate buffer at pH 6.0 (Dako, Glostrup, Denmark). The sections were incubated with the primary antibody against alpha smooth muscle actin (αSMA, 1:500 dilution, 1A4, Sigma-Aldrich, St. Louis, MO, USA) and CK19 (1:100 dilution, NBP1-53204, Novus Biologicals, Littleton, CO, USA) in phosphate buffer saline (PBS) overnight at 4 °C. After washing in PBS, the sections were incubated with secondary fluorescent antibody (Alexa Fluor 488 donkey anti-rabbit IgG, 1:100 dilution, A27034 & Alexa Fluor 594 goat anti-mouse IgG, 1:100, A11032, Thermofisher Scientific, Waltham, MA, USA) for 30 min at room temperature, before being washed in PBS and mounted with Fluoroshield™ with DAPI (Sigma-Aldrich) and imaged using Nanozoomer-RS (Hamamatsu Photonics, Hamamatsu, Japan).

### 2.2. Cell Culture

Pancreatic stellate cells (PSCs, ScienCell, Carlsbad, CA, USA) were cultured in stellate cell medium supplemented with 2 *v*/*v* % fetal bovine serum (FBS), 100 U/mL penicillin/100 µg/mL streptomycin (Pen/Strep) and 1 *v*/*v* % stellate cell growth supplement (SteCGS) according to manufacturer’s instruction (all products from ScienCell). Panc-1 cancer cells (ATCC, Manassas, VA, USA) were cultured in DMEM - High Glucose HyClone medium (GE Healthcare Life Sciences, Chicago, IL, USA) containing 10 *v*/*v* % FBS, 100 U/mL penicillin/100 µg/mL streptomycin (Thermofisher Scientific) and 2 mM L-glutamine (Thermofisher Scientific). The cells were maintained at 37 °C in a humidified 5% CO_2_ atmosphere and passed at 80% confluence. Passing of cells was performed as follows: The cells were washed twice with warm Dulbecco’s phosphate buffered saline (DPBS) (Lonza, Basel, Switzerland) before addition of trypsin/ethylenediaminetetraacetic acid (EDTA) (Thermofisher Scientific) and incubation at 37 °C. The trypsin/EDTA mix was neutralized by using 10× cell culture medium before being transported to a sterile Falcon tube and counted using a hemocytometer (Buerker-Tuerk, Brand GMBH, Wertheim, Germany). PSCs were used from a passage of 4–10.

### 2.3. Generation of Panc-1/PSC Utissues

Panc-1/PSC µtissues were generated by combining a spheroid culture with simple additive manufacturing techniques. In brief, Panc-1 spheroids were generated by culturing Panc-1 cancer cells (5000 cells/well) in a round-bottom 96 well plate that has been coated overnight with 1 *w*/*v* % pluronic^®^ F127 (Sigma-Aldrich) at 37 °C, washed with sterile MiliQ and air-dried. Before seeding, 2.5 *v*/*v* % Matrigel™ (Corning Inc., Corning, NY, USA) was added to the culture to promote cell aggregation and the formation of dense Panc-1 spheroids. The Panc-1 spheroid were allowed to form for 3 days of undisturbed culture at 37 °C.

Panc-1/PSC µtissues were prepared in a 3-step fabrication protocol: Firstly, PSCs (4 × 10^6^ PSCs/mL) were embedded in a collagen hydrogel (4 mg/mL), which was prepared using manufacturer’s instructions. In brief, collagen matrix (5 mg/mL, Matrix Biosciences, Mörlenbach, Germany) was mixed with 10× concentrated M199 medium, 1 N NaOH, 1 M 4-(2-hydroxyethyl)-1-piperazineethanesulfonic acid (HEPES) buffer and sterile water to achieve a pH of 7.4 (all products from Sigma-Aldrich). The PSC-laden hydrogel was transferred to custom-made PDMS (Dow Sylgard™ 184 Silicone Elastomer, Mavom BV, Alphen aan den Rijn, The Netherlands) microwells (Ø 3 mm, 3 mm height, Figure S1) to fill ½ of the well. The PSC-laden collagen gel was allowed to solidify at 37 °C for 1 h before proceeding. Secondly, the formed Panc-1 spheroid was placed onto the middle of the solidified collagen gel. Thirdly, the well was filled with PSC-laden collagen to the final height of 3 mm embedding the spheroid in the middle of the gel and allowed to solidify for 1 h at 37 °C before adding 50/50 culture medium (50% PSC culture medium/50% DMEM culture medium).

Panc-1 or PSC only µtissues were prepared in a similar way, however without PSCs in case of the Panc-1 only µtissues or without the Panc-1 spheroid in case of PSC only µtissues. Both conditions were cultured in the 50/50 culture medium.

All µtissues were allowed to contract for 5 days, while being photographically imaged on a daily basis to track the contraction.

### 2.4. Scanning Electron Microscopy (SEM) of Utissues

Panc-1/PSC µtissues and Panc-1 µtissues were allowed to contract for 5 days before being washed with DPBS and fixed with 2.5 *v*/*v* % glutaraldehyde (Electron Microscopy Sciences, Hatfield, PA, USA) for 1h at room temperature and at 4 °C overnight. The fixed µtissues were washed three times with MilliQ water before being frozen in liquid nitrogen. Afterwards the µtissues were lyophilized (TFD5503 Freeze Dryer, ilShin BioBase Europe, Ede, The Netherlands), gold-sputtered (Sputter Coater 108 Auto, Cressington Scientific Instruments, Watford, UK) and imaged using a scanning electron microscope (JSM-IT100, JEOL, Tokyo, Japan) at an accelerating voltage of 5 kV and a probe current of 35. The size of the µtissues was determined using ImageJ (Public, developed by Wayne Rasband (NIH)). The fiber diameter was calculated using Matlab (The Mathworks Inc., Natick, MA, USA).

### 2.5. Generation of Panc-1/PSC Heterospheroids

Panc-1/PSC heterospheroids were prepared similar to Panc-1 spheroids alone. In brief, Panc-1 and PSC were mixed prior to seeding at a ratio of 1:5 and cultured a round-bottom 96 well plate that has been coated overnight with 1 *w*/*v* % pluronic® F127 (Sigma-Aldrich) at 37 °C, washed with sterile MiliQ and air-dried. The heterospheroids were allowed to form for 3 days of undisturbed culture 37 °C. Due to the presence of PSCs no Matrigel™ was require for spheroid formation.

### 2.6. Immunostaining of Utissues and Heterospheroids

Panc-1/PSC µtissues and Panc-1/PSC heterospheroids were formed as previously described before being washed with DPBS, fixed with 4% formaldehyde (Sigma-Aldrich), washed again with DPBS and being embedded into Cryomatrix™ (Thermofisher Scientific) before being snap-frozen using isopentane (Sigma-Aldrich). Embedded and frozen µtissues and heterospheroids were cut into 6 µm thick cryosections, air-dried, and fixed with acetone for 10 min at room temperature. A circle was drawn around the tissues using a hydrophobic PAP pen (Sigma-Aldrich) before being rehydrated in PBS. The rehydrated sections were incubated overnight in primary antibody against CK19 (1:100 dilution, NBP1-53204, Novus Biologicals, Littleton, CO, USA) overnight at 4 °C. Next, the sections were washed with PBS, before being incubated with fluorescent secondary antibody (Alexa Fluor 594 donkey anti-rabbit IgG, 1:100 dilution, A-21207, Thermofisher Scientific), washed again in PBS and mounted using Fluoroshield™ with DAPI and imaged using Nanozoomer-RS.

### 2.7. Hematoxylin-Eosin (HE) Staining of Utissues and Heterospheroids

Cryosections of Panc-1 µtissues, Panc-1/PSC µtissues and Panc-1/PSC heterospheroids were prepared as previously described. After air-drying, the sections were fixed with 4 *v*/*v* % formaldehyde for 15 min, washed twice with MilliQ and incubated with hematoxylin (Sigma-Aldrich) for 15 min before being rinsed under tap water for 15 min. Next, the sections were incubated with eosin solution (Sigma-Aldrich) for 90 seconds before being washed in 96 *v*/*v* % ethanol. After washing, the sections were dehydrated using a series of 2 × 96 *v*/*v* % ethanol and 2 × 100% ethanol (1 min incubation). The dehydrated sections were mounted using DPX mounting for histology solution (Thermofisher Scientific) before being imaged using Nanozoomer-RS.

### 2.8. Gene Expression Profile of Utissues and Heterospheroids

Panc-1 µtissues, Panc-1/PSC µtissues and Panc-1/PSC heterospheroids were prepared as previously described. To allow the isolation of RNA from Panc-1 µtissues and Panc-1/PSC µtissues, the collagen hydrogel of the µtissues was degraded by incubation in a mixture of 3 mg collagenase (Sigma-Aldrich)/3 mg dispase II (Sigma-Aldrich) at 37 °C for 30 min. The resulting cell suspension was centrifuged to achieve a cell pellet before RNA isolation. For the Panc-1/PSC heterospheroids, at least 10 spheroids were pooled before RNA isolation. The total was isolated using the GenElute™ Mammalian Total RNA Miniprep Kit (Sigma-Aldrich) and the RNA amount was measured by a Nanodrop^®^ ND-1000 Spectrophotometer (Thermofisher Scientific). Next, cDNA was synthesized with iScript™ cDNA Synthesis Kit (BioRad, Veenendaal, The Netherlands). For each PCR reaction 10 ng cDNA was used. Abbreviation of all different genes in Table S1. All real-time PCR primers (Table S2) were purchased from Sigma-Aldrich. Quantitative real time PCR was performed with the 2× Sensimix SYBR and Flurescein Kit (Bioline GmBH, Luckenwalde, Germany) using a BioRad CFX384 real-time PCR detection system (BioRad). For 2D controls, Panc-1 cells and PSCs were cultured in conventional tissue culture-treated well plates (Cellstar^®^, Greiner Bio One) and cultured for 2 days before the total RNA was isolated, cDNA prepared, and PCR performed as mentioned above. For the comparison between 2D and 3D samples, all gene expression levels were normalized to expression of the house-keeping gene RPS18. For the comparison between Panc-1 µtissues, Panc-1/PSC µtissues and Panc-1/PSC heterospheroids all gene expression levels were normalized for CK19 in the case of Panc-1 and for FAP in the case of PSC, after confirming that these markers are exclusive for the respective cell type.

For the heat map, the average value of three independent experiment is displayed and the heat map is designed using Microsoft Excel (Microsoft, Redmond, WA, USA).

### 2.9. Transcriptomic Expression Analysis in Human Cohort from Public Database

A PDAC gene expression from the Expression Omnibus Database (GEO) was selected and downloaded. GSE15471 comprises of data from 36 PDAC patients, where each PDAC tissue and healthy adjunct pancreatic tissue, serving as control, was obtained from the same patient [24,25]. To access the expression of mRNA in gliomas versus control samples GEO2R was used, and the resulted normalized gene expression was plotted.

### 2.10. Flow Cytometry to Determine Panc-1 Cell Count & Analysis of Panc-1 Core Size

Prior to the generation of Panc-1 µtissues and Panc-1/PSC µtissues, Panc-1 spheroids were labelled with CellTracker™ Green CMFDA (Thermofisher Scientific). The µtissues were generated and cultured as previously described. Formed µtissues were pooled together (10 µtissues/experiment), washed twice with PBS and enzymatically degraded using 3 mg/mL collagenase and 3 mg/mL dispase. The amount of Panc-1 was determined using a BD FACS Calibur (BD Biosciences, Franklin Lakes, NJ, USA) and analyzed using Flowing Software 2 (Public, developed by Turku Bioscience). The average Panc-1 cancer cell count was determined by dividing the total count of Panc-1 cells by the number of µtissues that were pooled for the experiment.

The size of the Panc-1 core was determined using cryosectioned and HE stained Panc-1 µtissues and Panc-1/PSC µtissues, prepared as previously described. The size of the Panc-1 core was determined using ImageJ.

### 2.11. Treatment of Panc-1/PSC Utissues with AV3 & Silica Nanoparticle Penetration

Panc-1/PSC µtissues were prepared as previously described and incubated with 50 µM and 100 µM AV3 on the day of µtissues formation (day 0) and being refreshed 2 days post-formation. The size of the µtissues was tracked on a daily basis to detect inhibition of contraction in the treated samples. The size of the µtissues was determined using Image J.

For the penetration of nanoparticles, Panc-1 cells were labelled with CellTracker™ Green CMFDA prior to Panc-1/PSC µtissue formation as previously described. After formation of the µtissues, they were incubated with red-fluorescent silica nanoparticles of a 100 nm size (Sicastar^®^-RedF, Mikromod Partikeltechnologie GmbH, Rostock, Germany) at a concentration of 500 µg/mL for 24 h at 37 °C. The culture plate was placed on a shaking plate during nanoparticle incubation to increase particle influx into the µtissues. After incubation, the µtissues were pooled together, washed with PBS and enzymatically degraded using 3 mg/mL collagenase and 3 mg/mL dispase. The amount of double-positive stained Panc-1 (green and red fluorescent positive) was determined using a BD FACS Calibur and analyzed using Flowing Software 2. The relative uptake in Panc-1 was determined by dividing the amount of double positive (green^+^/red^+^) by the total amount of Panc-1 cells (green^+^).

### 2.12. Schematic and Statistical Analysis

All graphs were made using GraphPad Prism Vol.9 (GraphPad Software Inc., San Diego, CA, USA) based on calculations using Microsoft Excel. Schematics were made using Inkscape (Open-source vector graphics editor). All values are expressed as mean ± standard error of the mean (SEM). Statistical significance of the results was performed by two-tailed unpaired student’s *t*-test for comparison between two treatment groups. Significant difference was determined for a *p*-value of * *p* < 0.05, ** *p* < 0.01, and *** *p* < 0.001 respectively.

## 3. Results

### 3.1. Mimicking the Characteristics of PDAC in Patients Using a 3D Engineering Approach

**Figure 1:**
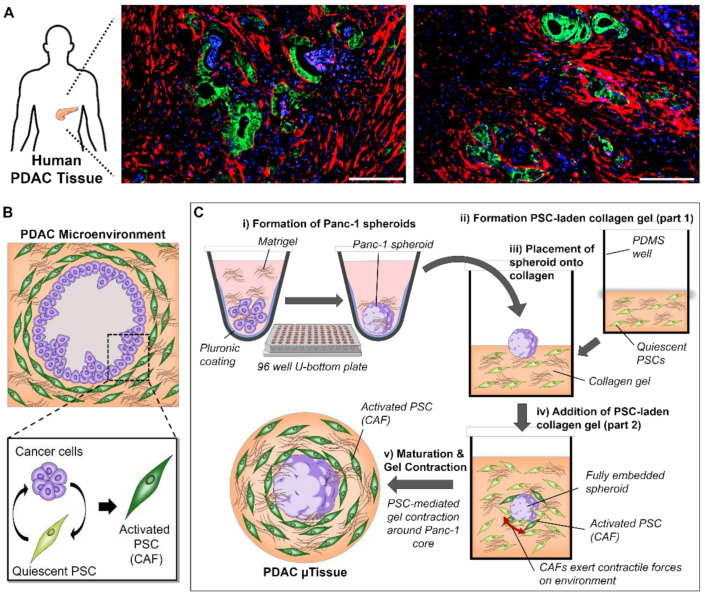
Cellular arrangement of PDAC and generation of PDAC µtissues. (**A**) Immunostaining for αSMA (red), CK19 (green) and nuclei (blue) in human PDAC tissues (scale bar = 200 µm). (**B**) Schematic representation of the cellular arrangement of pancreatic cancer cells and pancreatic stellate cells (PSCs) highlighting the crosstalk and subsequent activation of PSCs. (**C**) Generation of PDAC µtissues starting with (i) the generation of Panc-1 cancer cell spheroids based on the culture in a pluronic-coated 96 well U-bottom plate, (ii) the formation of a PSC-laden collagen gel and (iii) placement of Panc-1 spheroid onto the formed collagen layer before (iv) embedding by addition of a second half of the PSC-laden collagen gel. (v) Maturation of the µtissues including the PSC-mediated gel contraction.

With the aim to establish an engineered microtissue (µtissue) model that replicates the dense and fibrotic microenvironment found in PDAC, we first investigated the cellular arrangement of tumor cells and CAFs based on the immunostaining of tumor sections obtained from PDAC patients (Figure 1A). We observed that the pancreatic ducts, here identified by a high expression of CK19 (green), in PDAC are surrounded by a high amount of αSMA expressing cells, which in PDAC can be identified as CAFs [10,11]. Interestingly we also saw that the expression of αSMA is very high in direct contact with the pancreatic ducts seemingly forming a fibrotic capsule around the pancreatic ducts, which demonstrates that a direct contact between pancreatic cancer cells and surrounding cells in the microenvironment is a crucial characteristic in PDAC.

Based on these observations on the cellular arrangement in PDAC patients, we aimed to design a model that is able to replicate this distinctive arrangement and interactions of cells in the PDAC microenvironment. The strategy we present here is based on two-step biofabrication process (Figure 1B): First, we generate pancreatic cancer cell spheroids by culturing Panc-1 cancer cells in a round bottom 96-well plate, which has been coated with pluronic F127 to avoid cell adherence. Next, these spheroids are sandwiched between a collagen gel, presenting the most abundant extracellular matrix protein in PDAC, containing primary patient-derived pancreatic stellate cells (PSCs) and culture in custom made PDMS wells (Figure S1). The direct interaction between Panc-1 spheroids and PSCs will eventually cause PSCs to obtain an activated CAF-like state, which further causes these activated PSCs to contract the collagen hydrogel [10,11]. By providing a solid core, such as a spheroid, it is aimed that this contraction will be directed around the spheroid, subsequently embedding the spheroids in the gel surrounded by a dense fibrotic environment, similar to the situation in PDAC patients.

### 3.2. The Combination of Pancreatic Stellate Cells and Cancer Cells Cause Hydrogel Contraction and Utissue Formation

**Figure 2:**
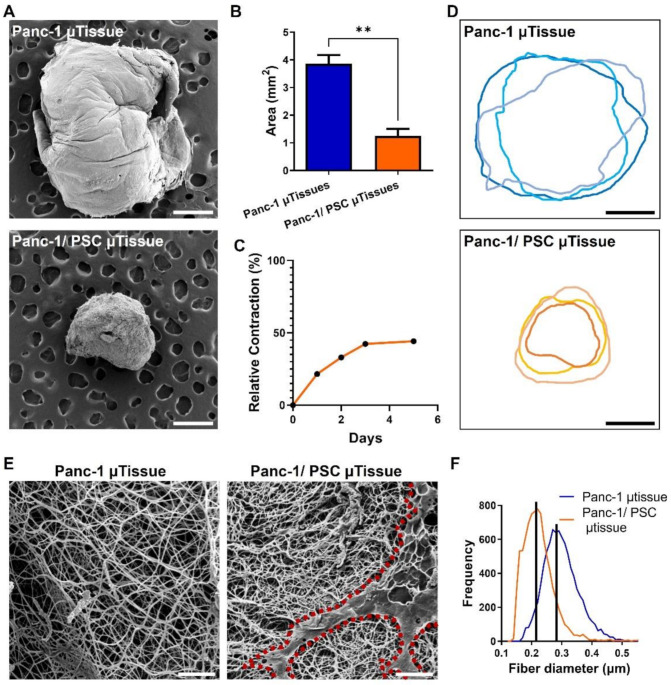
PSC-mediated contraction of PDAC µtissues. (**A**) Scanning electron microscopy (SEM) images for Panc-1 µtissues (top) and Panc-1/PSC µtissues (bottom) (scale bar = 500 µm). (**B**) Quantification of the µtissue size based on SEM images. (**C**) Relative contraction of Panc-1/PSC µtissues for a culture duration of 5 days. (**D**) Individual outlines for 3 individual Panc-1 µtissues (top) and Panc-1/PSC µtissues (bottom) (scale bar = 500 µm). (**E**) SEM images of the surface structure of Panc-1 µtissues (left) and Panc-1/PSC µtissues (right). Red outline highlighting PSC on the surface of hydrogel (scale bar = 10 µm). (**F**) Quantification of the fiber diameter based on SEM images. Data represent mean ± standard error of the mean for at least 3 independent experiments. Statistical analysis was performed by two-tailed Student’s *t*-test. ** *p* < 0.01.

The contractile properties of PSCs have been previously reported and can be directly related to an activated phenotype of PSCs [18,26,27]. To investigate whether the direct co-culture of Panc-1 spheroids and PSCs is indeed causing a contraction of the collagen hydrogel we cultured µtissues containing only Panc-1 spheroids and Panc-1/PSC µtissues and observed the difference in size after culture for 5 days. We found that, while µtissues of Panc-1 present an average size of ~3.8 ± 0.55 mm^2^, Panc-1/PSC µtissues achieve a final size of ~1.24 ± 0.44 mm^2^, hence a 3 times smaller size compared to Panc-1 alone (Figure 2A,B), confirming the contractile property of PSCs in the culture and also the potential activation of these PSCs in the model. Furthermore, we found that the contraction of the collagen hydrogel by PSCs mainly takes place in the first 72 h of culture achieving a maximum contraction of around 50% of the original size, while only minimally decreasing when cultured for additional 48 h (Figure 2C). Based on this, we were able to determine that an optimal culture period for these µtissues lies between 72–96 h. Moreover, we observed that, despite the fact that contraction is mainly driven by the PSCs themselves which might result in high variation between the samples, we were able to see a highly similar contraction profile for at least 3 individual experiments, confirming the reproducibility of the novel µtissues (Figure 2D).

Besides confirming that the µtissues are able to contract in general, were interested to investigate how the contraction changes the surface morphology of the collagen hydrogel. Using scanning electron microscopy, we observed that the surface of the Panc-1 µtissues alone, on the one hand, is porous in nature depicting the individual collagen fibers and larger space in between (Figure 2E). The combination of Panc-1/PSC on the other hand clearly demonstrates a denser environment with smaller space between the collagen fibers as well as the presence of PSCs on the hydrogel surface (indicated by red outline). To further confirm morphological changes in the collagen, we measured the fiber diameter of the individual collagen fibers and found a fiber diameter for the Panc-1/PSC µtissues compared to the Panc-1 µtissues alone, indicating a more contracted state of these fibers further confirming successful contraction of the whole microenvironment (Figure 2F).

### 3.3. The Novel 3D Engineered Microtissues Offer High Control on PDAC Relevant Cellular Arrangement

After confirming that the collagen hydrogel undergoes contraction based on the presence and potential activation of PSCs, we wanted to confirm that the collagen hydrogel is indeed contracting around the Panc-1 spheroid, creating a dense microenvironment with the Panc-1 spheroid in the center as seen in the in vivo situation. Furthermore, we were interested to demonstrate the advantage of the novel µtissues over a PDAC model that has been previously developed in our group consisting of the co-culture of Panc-1 cells and PSCs in a heterospheroid [15].

**Figure 3:**
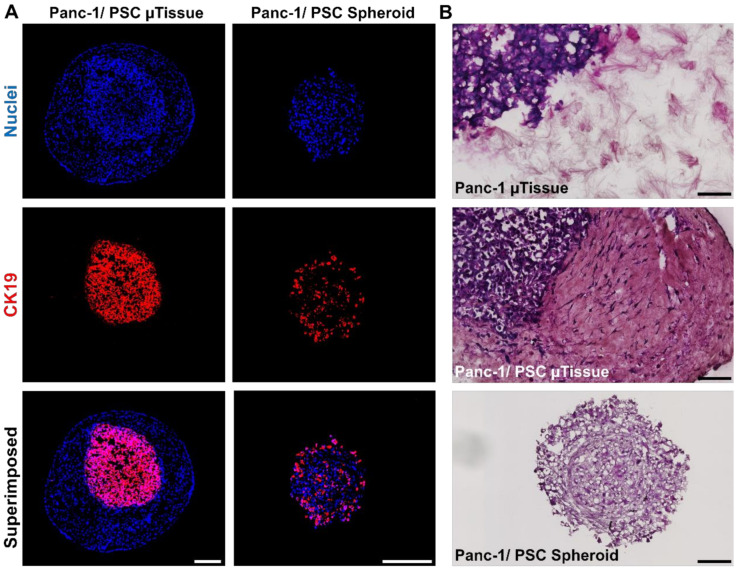
Cellular arrangement of Panc-1/PSC µtissues. (**A**) Immunofluorescent staining against CK19 (red) and nuclei (blue) for Panc-1/PSC µtissues and Panc-1/PSC heterospheroids (scale bar = 250 µm). (**B**) Hematoxylin-Eosin (HE) staining for Panc-1 µtissues, Panc-1/PSC µtissues and Panc-1/PSC heterospheroids (scale bar = 100 µm).

To confirm the cellular arrangement, we prepared cryosections of our novel Panc-1/PSC µtissues and of the conventional Panc-1/PSC heterospheroids and performed an immunostaining for CK19, a known marker for epithelial cells [28], to demonstrate the location of Panc-1 cells in the model (Figure 3A). We found that, in the Panc-1/PSC µtissues, the Panc-1 spheroid is successfully encapsulated by the collagen hydrogel containing PSCs demonstrated by the CK19^+^ only being present in the center of the model. The Panc-1/PSC heterospheroids, however, display a mixed population of Panc-1 cells and PSCs without a clear cellular arrangement, demonstrated by CK19^+^ being present throughout the whole spheroid. Furthermore, it appeared that high amounts of Panc-1 cells are located on the outer side of the spheroid, which is arguably not representing the in vivo situation realistically. The controlled location of Panc-1 cells in our novel µtissues is a clear advantage compared to the random distribution in the previous heterospheroid model and represents the realistic situation in a biologically relevant fashion.

Next, we investigated the interaction between the Panc-1 spheroid and its direct surrounding by performing a hematoxylin/eosin (HE) staining on the prepared cryosections and focus on the area of interaction between the different components of the model (Figure 3B). We observed that the Panc-1 spheroid only embedded in collagen hydrogel without PSCs (Panc-1 µtissues) is only loosely attached to the collagen surrounding, while the combination of Panc-1 spheroid and PSCs demonstrated a strong connection and adhesion between the Panc-1 spheroid and the surrounding without any noticeable gaps between the two areas. This confirms that the Panc-1 spheroid is fully integrated into the model and that cells are able to interact freely. In addition, we were able to again confirm the random distribution of cells in the Panc-1/PSC heterospheroids as we did not observe any clear morphological patterns or different areas in HE stained section of the heterospheroids, again demonstrating the structural advantage of our novel µtissues.

### 3.4. 3D Engineered Mictorissues Express a PDAC-Relevant Gene Profile

After demonstrating the morphological and structural advantage of our novel µtissues, we investigated the transcriptomic profile of the µtissues based on markers known for PDAC from literature to investigate the crosstalk of Panc-1 cells and PSCs in greater detail [17,28,29], and further compared it with the profile of the conventional heterospheroid culture. In addition, we further confirmed the expression of PDAC relevant genes within the µtissues by analyzing publicly available transcriptomics data of PDAC patients.

#### 3.4.1. 3D Microtissues Display Significant Upregulation of PDAC Specific Markers

We analyzed the transcriptomic profile of our novel µtissues in two different ways: (i) Comparing the expression of genes, that have been previously reported for PDAC [17,28,29], in 2D monolayer cultures of Panc-1 and PSC compared to the Panc-1 and PSCs cultured in the collagen hydrogel (Panc-1 and PSC µtissues, respectively) and (ii) compare the expression of the Panc-1 and PSC µtissues with Panc-1/PSC heterospheroids and Panc-1/PSC µtissues to demonstrate the advantage of our Panc-1/PSC µtissues compared to the single culture and heterospheroid culture.

First, we found that comparing the 2D monolayer cultures to the 3D µtissue cultures displayed a significant upregulation of several PDAC related genes in both, Panc-1 and PSC (Figure S2, full names for abbreviated genes can be found in Table S1). Interestingly, we first found that compared to the 2D monolayer culture, Panc-1 cells embedded in collagen displayed a significant upregulation of genes involved in ECM production (COL1α1, FN1, POSTN), ECM remodeling (MMP2), angiogenesis (VEGFα), expression of cytokines and surface receptors (IL-6, TGFβR1, PDGFRβ) as well as migration and EMT (αSMA, VIM), which demonstrated that a change of conformation from a 2D to a 3D environment already significantly alters cell behavior to be more biologically relevant. PSCs, on the other hand, also display a significant upregulation of markers involved in ECM production (COL1α1, FN1, POSTN, TN-C), ECM remodeling (MMP2) and general CAF markers (αSMA, TGFβR1, PDGFRβ, IL-6), showing that these cells also significantly react and adapt to the 3D environment. In particular, the upregulation of these markers indicates that PSCs in the given 3D environment are already activated towards a CAF-like phenotype based on autocrine signaling.

After comparing the 2D monolayer culture with the culture in a 3D collagen environment, we compared the expression of these markers when co-culturing Panc-1 and PSC in form of heterospheroids or µtissues compared to the culture of Panc-1 µtissues and PSC µtissues alone. To correct for the amount of the opposite cell type in the co-cultures, we corrected for Panc-1 with the expression of CK19 and for PSC with the expression of FAP, respectively, as these markers are exclusively expressed by these cell types (Figure S3).

**Figure 4:**
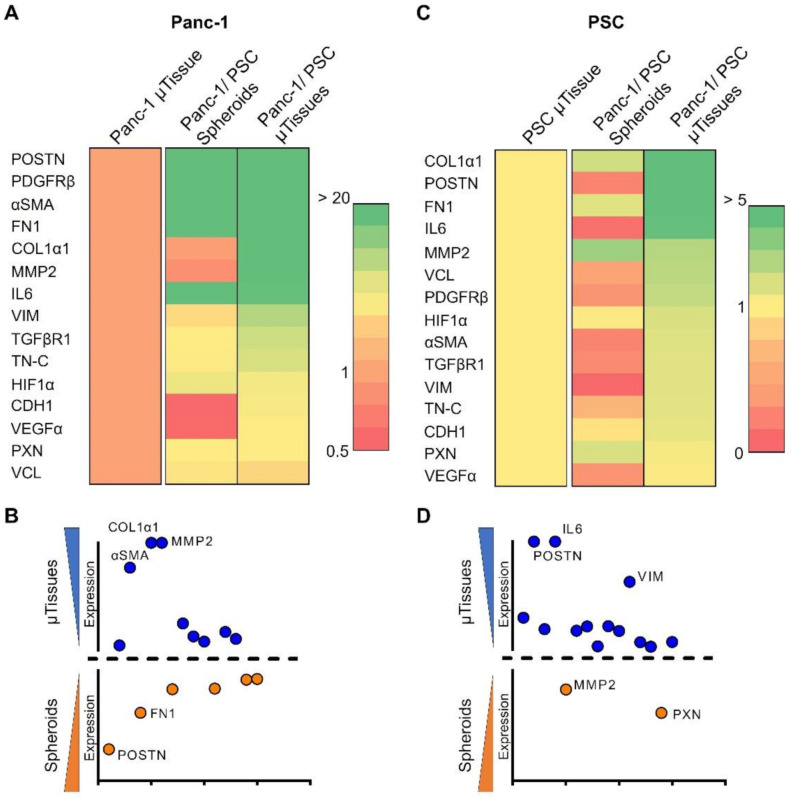
Gene profile of the interaction between Panc-1 cells and PSCs. (**A**) Heat map of expressed genes in Panc-1 cancer cells for Panc-1 µtissues, Panc-1/PSC heterospheroids and Panc-1/PSC µtissues. (**B**) Comparison of Panc-1 gene expression in Panc-1/PSC µtissues versus Panc-1/PSC heterospheroids. (**C**) Heat map of expressed genes in PSC for PSC µtissues, Panc-1/PSC heterospheroids and Panc-1/PSC µtissues. (**D**) Comparison of PSC gene expression in Panc-1/PSC µtissues versus Panc-1/PSC heterospheroids.

Remarkably, we found that Panc-1 cells demonstrated a nearly 3000 times higher expression of POSTN as well as a 917-, 137- and 6-times higher expression of FN1, COL1α1, and TN-C, respectively, in the Panc-1/PSC µtissues compared to the Panc-1 µtissues alone, which indicates a higher ECM production of Panc-1 in the co-culture condition (Figure 4A and Figures S4 and S5). Furthermore, a 33 times higher expression of MMP2 and 10 times higher expression of VIM demonstrate active matrix remodeling and potential epithelial-mesenchymal transition (EMT) of Panc-1 cells in the co-culture. Interestingly, we also observed 3 times increase in the expression of HIF1α, which indicates the presence of hypoxic cells due to the dense stroma surrounding the Panc-1 spheroid. When comparing the expression of these markers between Panc-1/PSC heterospheroids and Panc-1/PSC µtissues, we see a mixed expression of genes where 9/15 genes are higher expressed in the µtissues, and 6/15 genes display higher expression in the heterospheroids (Figure 4B). In particular, αSMA, COL1α1 and MMP2 are significantly higher expressed in the Panc-1/PSC µtissues (20 times, 140 times and 40 times higher expression, respectively), while especially POSTN and FN1 are higher expressed in the Panc-1/PSC heterospheroid (3.5 times and 1.6 times higher expression, respectively).

PSC in the co-culture, on the other hand, display a significant upregulation of ECM production markers such as COL1α1, POSTN, FN1, VCL and TN-C with an expression, which is 18, 10, 9.9, 2.7 and 1.7 times higher compared to the PSC µtissues alone, respectively (Figure 4C, Figures S4 and S5). Furthermore, we observed a 4.9 times higher expression of IL-6 and 1.8 times higher expression αSMA in the Panc-1/PSC µtissues indicating that the co-culture of PSCs with Panc-1 cancer cells is causing a more activated state of PSCs compared to the autocrine activation of PSCs alone as previously mentioned. Remarkably, we found that when comparing the expression in Panc-1/PSC µtissues and Panc-1/PSC heterospheroids, we observed a significantly higher expression of nearly all genes in the µtissues. In detail, 13/15 genes are higher expressed in the Panc-1/PSC µtissues of which POSTN, IL-6 and VIM display the highest upregulation with 34-, 28- and 16-times higher expression compared to the heterospheroids (Figure 4D). Only MMP2 and PXN displayed a slightly higher expression in the Panc-1/PSC heterospheroids with an expression that is 1.2 times and 1.6 times higher in the heterospheroids compared to the µtissues. These results clearly demonstrate that, especially for PSCs behavior, the µtissues offer a beneficial platform due to the higher expression of PDAC relevant genes in this environment.

#### 3.4.2. Genes Expressed in 3D Microtissues Also Display a Significant Upregulation in PDAC Patients

**Figure 5:**
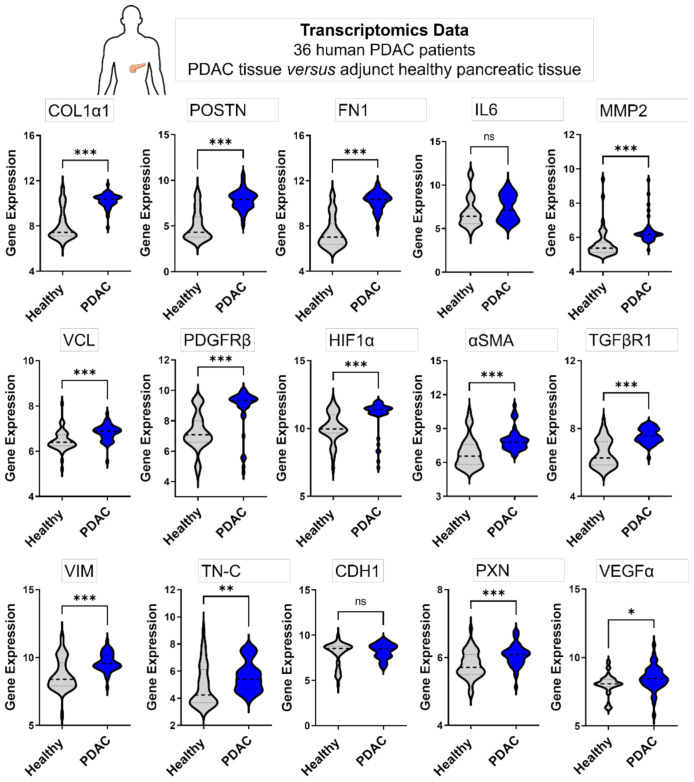
Comparative transcriptomic analysis in human PDAC patients. Gene expression in healthy adjunct pancreatic tissue and PDAC tissue from the same patient (36 PDAC patients in total). Statistical analysis was performed by two-tailed Student’s *t*-test. * *p* < 0.05, ** *p* < 0.01, *** *p* < 0.001.

To confirm that the genes upregulated in our Panc-1/PSC µtissues are of relevance to the gene profile of PDAC patients, we performed a transcriptomic analysis of publicly available patient data. Data from 36 PDAC patients was analyzed, where each PDAC tissue and healthy adjunct pancreatic tissue, serving as control, was obtained from the same patient [24,25]. We found that nearly all genes that were reported in literature to play a crucial role in PDAC are indeed found significantly upregulated in PDAC tissues compared to the healthy control (Figure 5). In particular genes that also display a crucial role and significant upregulation in our Panc-1/PSC µtissues such as COL1α1, POSTN, FN1, MMP2 or αSMA. Moreover, VCL, PDGFRβ, TGFβR, and VIM also show a highly significant upregulation in PDAC tissues. The upregulation of these genes in patients and in our Panc-1/PSC µtissues demonstrates the importance of the chosen genes. Interestingly, we found that IL-6, a marker that shows high upregulation in our Panc-1/PSC µtissues, does not display an upregulation in patients. This difference might display certain limitations of our novel model but also indicates the expression of IL-6 might be influenced by other ECM components which dilute the expression causing the lack of upregulation of this marker in PDAC tissues [30,31]. By implementing more cells found in the PDAC microenvironment such as macrophages and by increasing the complexity of our Panc-1/PSC µtissues, it might be possible to further increase the understanding of the different functions of the different PDAC microenvironment components and to determine the contribution of each component to the overall genetic profile of PDAC.

### 3.5. The Co-Culture of Pancreatic Stellate Cells and Cancer Cells Significantly Increases Cancer Cells Proliferation

The interaction of Panc-1 and PSC does not solely affect PSCs, which attain an activated and contractile phenotype, but also effects Panc-1 cells. As we have shown in the previous section that Panc-1 display increased migratory properties based on the expression of vimentin, we were interested to investigate the effect of PSC co-culture on the proliferation Panc-1 cells in our Panc-1/PSC µtissues.

**Figure 6:**
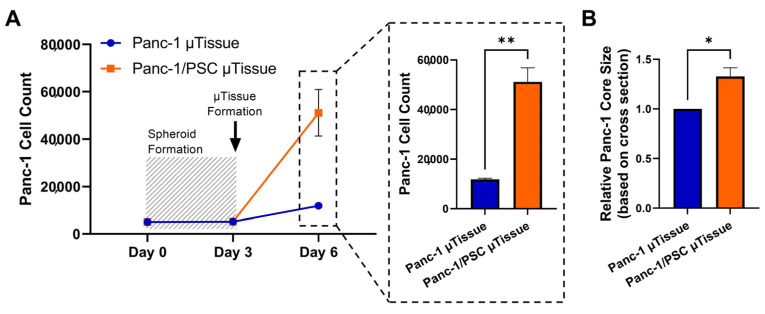
Proliferation of Panc-1 cells in Panc-1/PSC µtissues. (**A**) Panc-1 cell count in Panc-1 µtissues and Panc-1/PSC µtissues determined by flow cytometry. (**B**) Size of Panc-1 core in Panc-1 µtissues and Panc-1/PSC µtissues based on HE stained tissue sections (relative size calculated from the measured cross-section area in mm^2^). Data represent mean ± standard error of the mean for at least 3 independent experiments. Statistical analysis was performed by two-tailed Student’s *t*-test. * *p* < 0.05, ** *p* < 0.01.

First, we investigated the number of Panc-1 cells in the Panc-1/PSC µtissues and Panc-1 µtissues alone serving as control. Here, we labelled Panc-1 cells with CellTracker Green CMFDA before spheroid generation, starting with 5000 cells/spheroids, and analyzed the number of Panc-1 cells after spheroid formation (day 3) and after 3 additional days of culture in the collagen hydrogel with and without PSCs (day 6) using flow cytometry (Figure 6A). We found that the number of Panc-1 cells did not significantly increase during spheroid formation, indicating that the cells have limiting growth when forming a spheroid, however, Panc-1 cells start to significantly proliferate when placed in the collagen environment. In particular, the number of Panc-1 cells in the Panc-1/PSC µtissues drastically increased from ~ 5200 cells to more than 50,000, while Panc-1 spheroids place in collagen without PSCs only increase to ~12,000, displaying a nearly 5 times increase in proliferation when Panc-1 are co-cultured with PSC.

To further confirm the increase in proliferation of Panc-1, we measured the area covered by the Panc-1 spheroid in collagen alone and in combination with PSCs based on performed HE staining. Interestingly we found that despite the dense and confining environment in the Panc-1/PSC µtissues, the Panc-1 spheroid in these cultures is roughly 1.4 times larger compared to the Panc-1 tissues alone (Figure 6B).

The higher proliferation of Panc-1 cells in the co-culture Panc-1/PSC µtissues clearly demonstrate the benefits of the co-culture model and the biological relevance of the model.

### 3.6. Inhibition of Integrin α5 (ITGA5) can Significantly Inhibit Microtissue Contraction and Increase Drug Penetration across Stromal Barrier

**Figure 7:**
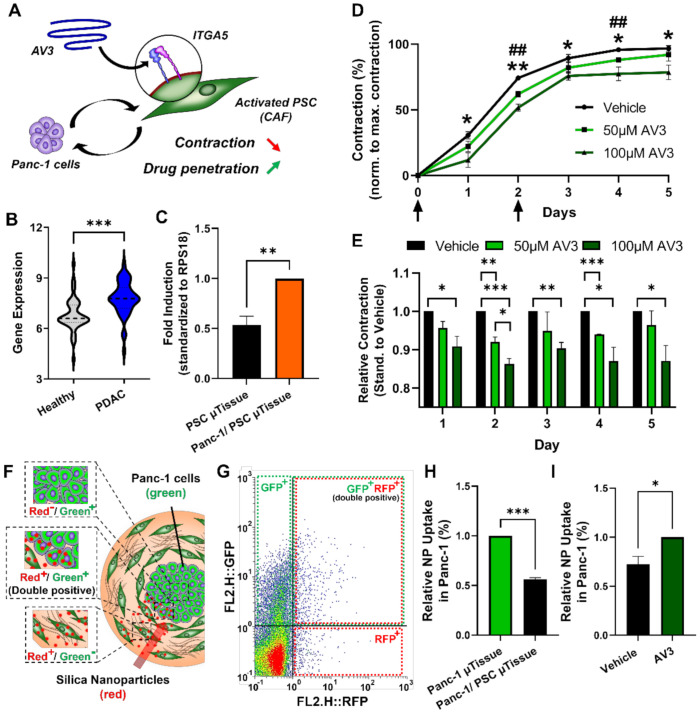
Treatment of Panc-1/PSC µtissues with ITGA5 antagonist AV3. (**A**) Schematic representation of the mechanism of action of AV3. (**B**) Expression of ITGA5 in human PDAC patients. (**C**) Expression of ITGA5 in Panc-1/PSC µtissues compared to PSC µtissues alone. (**D**) Size of Panc-1/PSC µtissues treated with 50 µM and 100 µM AV3 for a culture duration of 5 days. Arrows indicating days of treatment. * indicates significance comparing treatment with vehicle versus 100 µM AV3. # indicates significance comparing treatment with vehicle versus 50 µM AV3. (**E**) Relative contraction of Panc-1/PSC µtissues treated with 50 µM and 100 µM AV3 for each day of culture. (**F**) Schematic representation of the experimental setup demonstrating the penetration of red fluorescent silica nanoparticles into Panc-1/PSC µtissues containing green fluorescent-labelled Panc-1 cancer cells, highlighting cells only positive for nanoparticles (red^+^/green^−^), positive for both, nanoparticles and green-labeling (double positive red^+^/green^+^) and cells only positive for the green-labeling (red^−^/green^+^). (**G**) Dot plot of flow cytometry analysis highlighting the different subpopulations in the Panc-1/PSC µtissues. (**H**) Relative number of double-positive cells in Panc-1 µtissues versus Panc-1/PSC µtissues. (**I**) Relative number of double-positive cells in Panc-1/PSC µtissues treated vehicle (DMSO) or 50 µM AV3 (same Panc-1/PSC µtissues were used for data presented in (**H**,**I**)). Data represent mean ± standard error of the mean for at least 3 independent experiments. Statistical analysis was performed by two-tailed Student’s *t*-test. * *p* < 0.05, **^, ##^ *p* < 0.01, *** *p* < 0.001.

After demonstrating the biological relevance of our novel Panc-1/PSC µtissues in terms of cellular arrangement, cell behavior and gene profile, we were interested in demonstrating the capability of our novel model to serve as a tool for drug testing. Recently, we demonstrated the inhibition of PSCs and related contraction using an integrin α5 (ITGA5) antagonistic peptide called AV3 (Figure 7A) [19]. ITGA5 is mainly involved in PSC binding to the ECM, in particular fibronectin, as well as plays a role in the activation of PSCs. By inhibiting these processes PSC contraction and therefore the contraction of our Panc-1/PSC µtissues should be inhibited enabling increased influx of drug molecules.

First, we investigated if ITGA5 does play an important role in PDAC by analyzing the ITGA5 expression in PDAC tissues compared to healthy adjunct pancreatic tissues using the publicly available transcriptomic dataset mention above. We indeed found that ITGA5 is significantly higher expressed in PDAC tissues compared to healthy pancreatic tissues confirming the potential of ITGA5 as clinical target (Figure 7B). We then examined if ITGA5 is also expressed in our Panc-1/PSC µtissues and found a significant upregulation in the Panc-1/PSC µtissues compared to the PSC µtissues alone, confirming that also in our model ITGA5 forms a suitable therapeutic target for drug testing (Figure 7C).

After confirming the expression of ITGA5 in our novel Panc-1/PSC µtissues, we incubated the µtissues with 50 µM and 100 µM AV3 on the day of µtissue generation (day 0) and repeated the treatment 2 days post-formation (day 2), while following the µtissue contraction on a daily basis. We found that, despite the general contraction of all tissues, 50 µM and 100 µM AV3 can significantly inhibit the contraction of the µtissues with an inhibition of 5–18% for 50 µM and 100 µM AV3, respectively (Figure 7D,E). Interestingly we see that at later culture days (days 3–5) the µtissues treated with 100 µM AV3 hardly contract at all but stagnate at a certain size, indicating that PSCs are successfully inhibited for a longer duration of time. This inhibition of contraction is in line with our previous findings demonstrating that AV3 can reduce the collapse of blood vessels in PDAC animal models allowing for higher drug penetration [19].

Based on the inhibition of contraction we were interested to investigate if this inhibition might also increase nanoparticle penetration. Here, we investigated the penetration of red fluorescent 100 nm silica nanoparticles into the Panc-1/PSC µtissues, where Panc-1 cells were labelled with green fluorescent CellTracker green CMFDA (Figure 7F). To determine the number of nanoparticles that are able to reach the Panc-1 tumor core and taken up by these cells, we measured the number of Panc-1 cells that are double positive, expressing green as well as red fluorescence (green^+^/red^+^) relative to the total amount of Panc-1 (green^+^), by flow cytometry. First, we found that out of all nanoparticles added, around 21.3% are able to enter the Panc-1 spheroid core in Panc-1 µtissues that did not include PSCs, while only 11.9% of the added particles can reach the Panc-1 core in the Panc-1/PSC µtissues showing a relative reduction of 43.7% of nanoparticle uptake, indicating the formation of strong barrier around the Panc-1 core similar to the situation found in vivo (Figure 7H and Figure S6A). Furthermore, we found that the treatment with AV3 significantly increased the penetration of nanoparticles from 11.9% for the Panc-1/PSC µtissues to 17% for Panc-1/PSC µtissues incubated with 50 µM AV3, displaying a relative increase of 29% (Figure 7I and Figure S6B). Although the overall penetration of nanoparticles is still lower compared to Panc-1 µtissues alone, treatment with AV3 increased the overall penetration by 29% compared to the untreated µtissue, which in the clinics might make a significant difference in patient care.

## 4. Discussion

Dense and highly fibrotic stroma in PDAC remains one of the main reasons for the poor therapeutic efficacy and low patient survival in the clinic. In this study, we designed a novel 3D PDAC µtissue model which replicates the specific cellular organization of the stroma and the tumor cells as it is found in PDAC tissues in patients. We demonstrated how the co-culture of Panc-1 and PSCs triggers the activation of PSCs and induces the contraction of the collagen hydrogel around the Panc-1 tumor cell core, resulting in Panc-1 being surrounded by a dense fibrotic environment as found in vivo. Our technique displayed high control on the cellular arrangement with a simple fabrication process. Furthermore, our novel 3D PDAC µtissues displayed a PDAC-relevant transcriptomic profile, which was in line with publicly available patient data. Moreover, we confirmed that the interaction between Panc-1 and PSCs induced Panc-1 cell proliferation and further demonstrated that our 3D PDAC µtissues can be used to evaluate novel therapeutics that aim to modulate the TME and for studying penetration and tumor uptake of nanoparticles in the presence of dense stromal barrier.

To the best of our knowledge, this is the first proof-of-concept 3D PDAC µtissue model consisting of a cancer cell core surrounded by primary PSCs generated by a simple 2-step method involving spheroid and additive manufacturing technologies that fully relies on the cells own ability to contract around a given core. Compared to the current techniques to fabricate 3D in-vitro models we offer a simple and reproducible fabrication technique that achieves a high control on the cellular composition, however, does not require special equipment for fabrication. In such a way, the proposed PDAC µtissue model is available for a broad audience. Furthermore, we demonstrated the potential culture of up to 165 µtissues displaying a high-throughput and cost-effectiveness, which might allow for a rapid screening of novel therapeutics. In such way, our novel PDAC µtissues might help in the reduction and the refinement of animal experiments.

For the fabrication of the µtissues, collagen was chosen as hydrogel as it has been previously demonstrated to allow the culture of PSCs in a 3D biologically relevant environment that allows cell contraction. Furthermore, collagen forms the most abundant protein in the PDAC ECM making it the most suitable base for mimicking the PDAC TME [1]. Besides collagen, however, other ECM proteins are also present in the PDAC TME such as fibronectin, periostin, vinculin or tenascin-C. We have shown the upregulated gene expression of these ECM proteins in our µtissues indicating that PSCs themselves produce these proteins similar to the situation in vivo.

The activation of PSCs towards a CAF-like phenotype is the underlying mechanism to form the µtissues and achieve an in vitro model that resembles the PDAC TME. While CAFs can be originated from different sources including infiltrated cells, PSCs form the major source of CAFs in the PDAC TME [1,32,33]. A different strategy would be the use of CAFs derived from patients directly, however, such cells are often difficult to obtain and challenging to maintain stable in in vitro cultures, making PSCs the ideal cell type to use in our µtissues [34]. Moreover, in the recent years different subtypes of CAFs have been identified including myofibroblast-like CAFs (myCAFs), represented by a high expression of αSMA, and inflammatory CAFs (iCAFs), represented by a high expression of IL6 [20,35]. It has been shown the proximity of CAFs to cancer cells is crucial for the activation towards my- or iCAF, where CAFs in direct juxtacrine interaction with cancer cells are presenting myCAF features while CAFs in distant paracrine interaction present iCAF characteristics [20,35,36,37]. In our µtissues we observed a particularly high expression of IL6 which was not present in Panc-1/PSC heterospheroids next to the expression of αSMA, which was present in both models. This indicates that the higher spatial control in our µtissues allows for the presence of different CAF subtypes in our µtissues. In particular the presence of iCAF can be explained by the central core of Panc-1 that allows juxtacrine interaction with PSCs, resulting in myCAF, but also paracrine interaction resulting in iCAF. In conventional Panc-1/PSC heterospheroids, only juxtacrine interaction is possible, demonstrating the advantages of our novel platform to mimic the realistic situation.

Despite the high abundance of PSCs in the PDAC TME, other cellular components also play a crucial role in the progression and treatment-resistance of PDAC. In particular, tumor-associated macrophages (TAMs) have recently shown to play a major function in PDAC [6,38,39]. We envision including such TAMs in the future to increase the complexity of our model in a controlled manner and to render our model even more biologically relevant. In addition, the inclusion of other immune cells such as natural killer cells or T cells together with TAMs might allow to achieve a model that is capable to replicate the PDAC immune environment allowing for the evaluation of novel immunotherapies in a biologically relevant environment. Moreover, the inclusion of endothelial cells might allow for the formation of blood-vessel like structures within the µtissues allowing to study angiogenesis in PDAC or the interaction between stromal components and endothelial cells [40]. Furthermore, the formation of blood vessel-like structures might facilitate the supply with nutrients and oxygen allowing for longer culture durations [41].

In particular, when increasing the complexity of the model with multiple cell types, a proper supply with nutrients and oxygen is crucial to achieve optimal culture conditions. We envision that in such way, cellular interactions and the efficacy of therapeutics can be studied over a prolonged time, which might help to develop novel therapeutics for the clinical applications.

Finally, we demonstrated the formation of stromal barrier surrounding the tumor core. This stromal barrier is a key issue for many therapies including nanomedicine [42]. Most nanomedicines are not able to penetrate through this barrier and reach the target cancer cells. As a result, novel strategies are in development to overcome this barrier and allow for increased treatment efficacy [19,43]. To develop stroma-penetrating therapies, it is crucial to develop models that incorporate such a stromal barrier to access therapy efficacy in a realistic environment. The combination of a CAF-modulating therapy in combination with a nanomedicine demonstrates that our µtissues form a suitable platform to evaluate such novel nanomedicine strategies in a biologically relevant fashion. Furthermore, it is envisioned that this platform can be used to predict the efficacy of conventional chemotherapy. As novel 3D models have shown promising capability to aid in drug development by allowing the optimization of treatment strategies, e.g., the combination of chemotherapy with a CAF-modulating therapies [19], our novel µtissues might also find application in the optimization of treatment strategies including chemotherapy before embarking on animal models. This might further render treatment strategies more efficient as well as reduce the optimization time and costs.

## 5. Conclusions

In conclusion, this study demonstrated the generation of novel 3D PDAC µtissues, which include the characteristic fibrotic environment of PDAC forming a dense and hard-to-penetrate barrier around the tumor cells. While using the cell-own contractility of activated PSCs, the model allowed for a high reproducibility and high throughput which makes the µtissues highly interesting for a broad audience that aims to use biologically relevant 3D models in their drug development.

Altogether, the realistic biomimetic characteristics of novel PDAC µtissues and the suitability to investigate different kind of therapies, such as TME modulating drugs, and the combination of different treatments demonstrate the use of our PDAC µtissues to serve as a platform for the development and rapid evaluation of novel treatment strategies against PDAC.

## Data Availability

We analyzed publicly available transcriptomic patient data. The dataset GSE15471 comprises of 36 PDAC patients, where each PDAC tissue and healthy adjunct pancreatic tissue, serving as control, was obtained from the same patient (https://www.ncbi.nlm.nih.gov/geo/query/acc.cgi?acc=gse15471 (accessed on 28 September 2021)) [24,25].

## Supplementary Materials

The following are available online at https://www.mdpi.com/article/10.3390/cancers13195006/s1, Figure S1: Custom-made PDMS well plates, Figure S2: Gene expression for 2D monolayer cultures versus 3D cultures, Figure S3: Specific expression of CK19 and FAP in Panc-1 and PSC 3D cultures, Figure S4: Gene expression profile with exact numbers, Figure S5: Gene expression comparing 3D single culture of Panc-1, Panc-1/PSC heterospheroids and Panc-1/PSC µtissues. Figure S6: Nanoparticle Uptake in Panc-1 cell core, Table S1: Gene abbreviations, Table S2: Primers used in real-time PCR.
