# Supplementary material for: Novel 3D µtissues Mimicking the Fibrotic Stroma in Pancreatic Cancer to Study Cellular Interactions and Stroma-Modulating Therapeutics
Source: Cancers (Basel). 2021 Oct 6;13(19):5006. doi: 10.3390/cancers13195006 (PMC8508009; doi:10.3390/cancers13195006)
Supplement: Supplementary file 1 [file cancers-13-05006-s001.zip › cancers-1359996-supplementary.pdf]

cancers

MDPI

*Supplementary Information*

# Novel 3D μtissues Mimicking the Fibrotic Stroma in Pancreatic Cancer to Study Cellular Interactions and Stroma-Modulating Therapeutics

**Kunal Pravin Pednekar [1#], Marcel Alexander Heinrich [1#], Joop van Baarlen[2] and Jai Prakash [1,*]**

1 Engineered Therapeutics group, Department of Biomaterials Science and Technology, Technical Medical Centre, University of Twente, 7500AE Enschede, The Netherlands
2 Laboratorium Pathologie Oost-Nederland (LabPON), Hengelo, The Netherlands
k.p.pednekar@utwente.nl
m.a.heinrich@utwente.nl
j.vanbaarlen@labpon.nl

* Correspondence: j.prakash@utwente.nl ; [#] Authors contributed equally

**Supplementary Table S1: Gene abbreviations.**

| Gene Abbreviation | Full Name |
| --- | --- |
| αSMA | Alpha smooth muscle actin |
| COL1α1 | Collagen, type 1, alpha 1 |
| FN1 | Fibronectin 1 |
| VIM | Vimentin |
| CDH1 | Cadherin 1 (E-cadherin) |
| TGFβR1 | Transforming growth factor beta receptor 1 |
| VEGFα | Vascular endothelial growth factor alpha |
| PDGFRβ | Platelet-derived growth factor receptor beta 1 |
| POSTN | Periostin |
| ITGA5 | Integrin alpha 5 |
| PXN | Paxillin |
| VCL | Vinculin |
| TN-C | Tenascin-C |
| HIF1α | Hypoxia-inducible factor 1 alpha |
| MMP2 | Matrix metallopeptidase 2 |
| IL-6 | Interleukin 6 |
| CK19 (also known as KRT19) | Cytokeratin 19 |
| FAP | Fibroblast activation protein |
| RPS18 (housekeeping) | Ribosomal protein 18 |

**Supplementary Table S2: Primers used in real-time PCR.**

| Gene | Forward Primer | Reverse Primer |
| --- | --- | --- |
| αSMA | CCCCATCTATGAGGGCTATG | CAGTGGCCATCTCATTTTCA |
| COL1α1 | GTACTGGATTGACCCCAACC | CGCCATACTCGAACTGGAAT |
| FN1 | GTATACGAGGGCCAGCTCAT | CCCAGGAGACCACAAAGCTA |
| VIM | TCCAGAGAGAGGAAGCCGAA | AAGGTCAAGACGTGCCAGAG |
| CDH1 | AACCCAAGCACGTATCAGGG | GAGTGTTGGGGGCATCATCA |
| TGFβR1 | TCTTGGAACCTGCTCTCCTG | GTCCCACAACTTCCATCAGC |
| VEGFα | GCTCAGAGCGGAGAAAGCAT | GCAACGCGAGTCTGTGTTTT |
| PDGFRβ | AGGCAAGCTGGTCAAGATCT | GCTGTTGAAGATGCTCTCCG |
| POSTN | ACAAGAAGAGGTCACCAAGGTC | CTTGCAACTTCCTCACGGGT |
| ITGA5 | CAACTTCTCCTTGGACCCCC | GTCCTCTATCCGGCTCTTGC |
| PXN | CATGGACGACCTCGACGC | CAAGAACACAGGCCGTTTGG |
| VCL | CAGAACTGGCTTGCAGATCC | CTCCTTTCCCCTGTCTTCGT |
| TN-C | GAAGAGCATTCCTGTCAGCG | CCCACTCCACTTCCACAGAT |
| HIF1α | ATGTAATGCTCCCCTCACCC | CAGGGTCAGCACTACTTCGA |
| MMP2 | TTTCTATGGCTGCCCCAAGG | GTCAAGGTCACCTGTCTGGG |
| IL-6 | TGATGCTGGTGACAACCACGGC | TAAGCCTCCGACTTGTGAAGTGGTA |
| CK19 | CGCGACTACAGCCACTACTA | AGAGCCTGTTCCGTCTCAAA |
| FAP | GCAGTGTATCGAAAGCTGGG | AGGGCCAGTGATGAAACGTA |
| RPS18 | TGAGGTGGAACGTGTGATCA | CCTCTATGGGCCCGAATCTT |

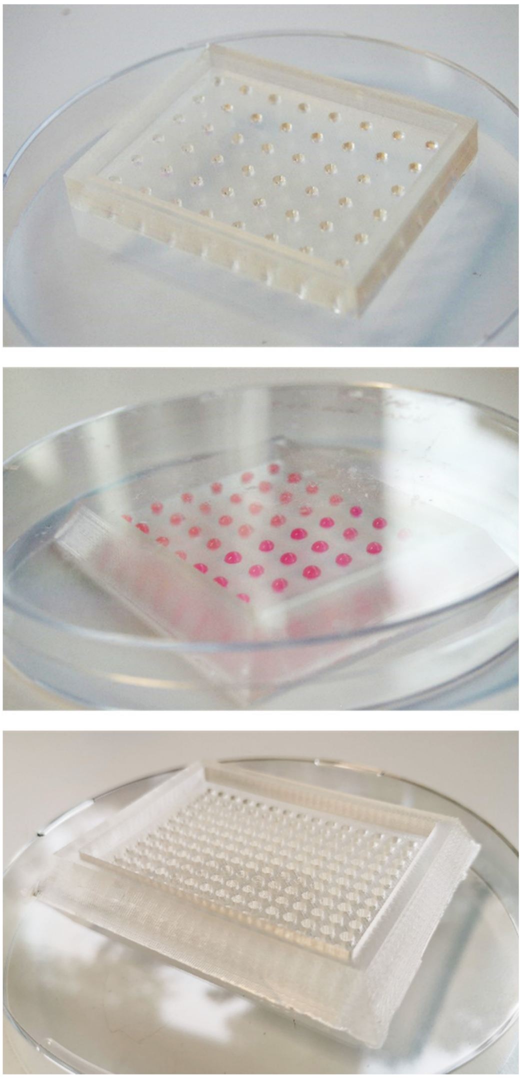

**Figure S1: Custom-made PDMS well plates.**

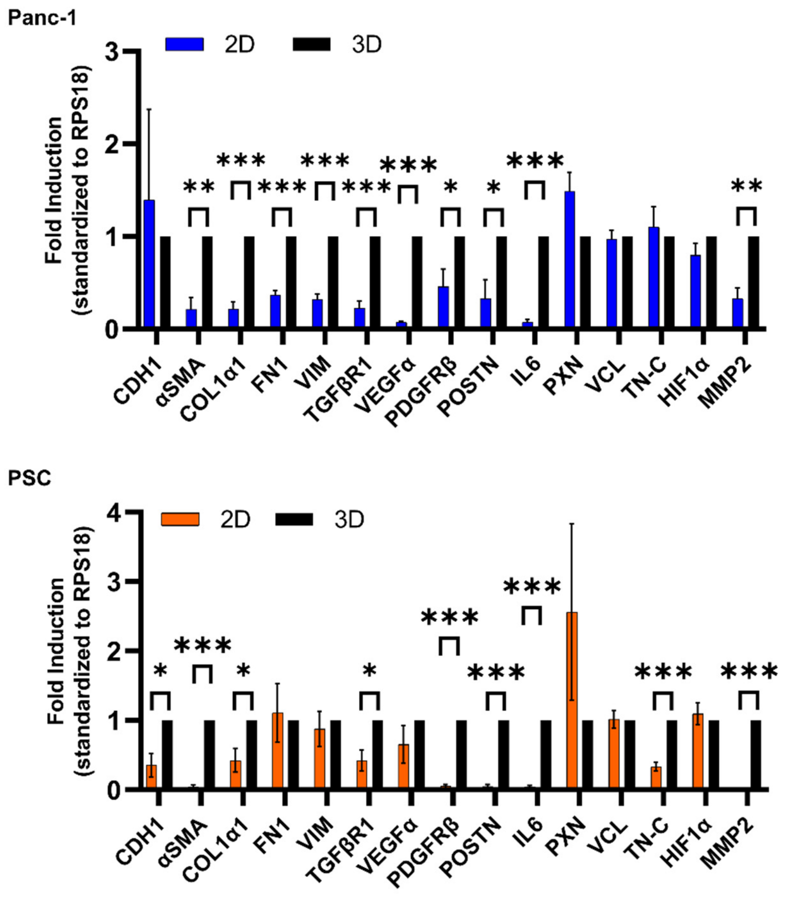


**Figure S2: Gene expression for 2D monolayer cultures versus 3D cultures.** Statistical analysis was performed by two-tailed Student's *t*-test. *$p < 0.05$, **$p < 0.01$, ***$p < 0.001$.

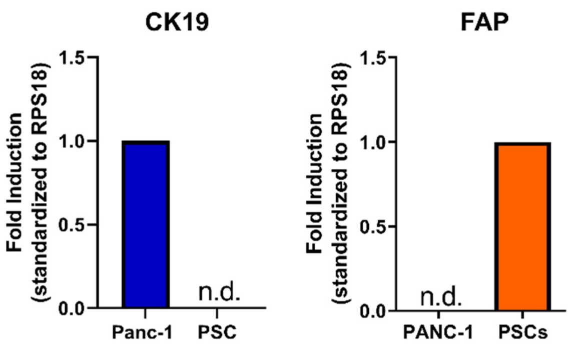


**Figure S3: Specific expression of CK19 and FAP in Panc-1 and PSC 3D cultures.**

**Panc-1**

| | Panc-1 µTissue | Panc-1/ PSC Spheroids | Panc-1/ PSC µTissues |
|---|---|---|---|
| POSTN | 1 | 10670.4 | 3058.136 |
| PDGFRβ | 1 | 1308.662 | 2859.181 |
| ACTA2 | 1 | 52.90958 | 1031.916 |
| FN1 | 1 | 1485.995 | 917.0333 |
| COL1α1 | 1 | 0.977361 | 137.9807 |
| MMP2 | 1 | 0.842493 | 33.16444 |
| IL6 | 1 | 23.55897 | 19.60408 |
| VIM | 1 | 1.475845 | 10.44948 |
| TGFβR1 | 1 | 1.830851 | 7.655798 |
| TN-C | 1 | 2.019891 | 6.150761 |
| HIF1α | 1 | 3.756505 | 3.151385 |
| CDH1 | 1 | 0.50648 | 2.613294 |
| VEGFα | 1 | 0.530613 | 1.923361 |
| PXN | 1 | 1.768136 | 1.627317 |
| VCL | 1 | 1.558485 | 1.444101 |

| |
|---|
| 20 |
| 15 |
| 10 |
| 7.5 |
| 5 |
| 2.5 |
| 1.25 |
| 1 |
| 0.8 |
| 0.6 |
| 0.5 |

**PSC**

| | PSC µTissue | Panc-1/ PSC Spheroids | Panc-1/ PSC µTissues |
|---|---|---|---|
| COL1α1 | 1 | 2.217327 | 18.13842 |
| POSTN | 1 | 0.299006 | 10.04996 |
| FN1 | 1 | 1.776217 | 9.981029 |
| IL6 | 1 | 0.17308 | 4.923643 |
| MMP2 | 1 | 3.478194 | 2.875902 |
| VCL | 1 | 0.526974 | 2.763158 |
| PDGFRβ | 1 | 0.414906 | 2.574895 |
| HIF1α | 1 | 1.113096 | 2.005294 |
| ACTA2 | 1 | 0.297566 | 1.864036 |
| TGFβR1 | 1 | 0.347521 | 1.820489 |
| VIM | 1 | 0.112112 | 1.805876 |
| TN-C | 1 | 0.632633 | 1.716668 |
| CDH1 | 1 | 0.944998 | 1.6475 |
| PXN | 1 | 2.016267 | 1.237862 |
| VEGFα | 1 | 0.407759 | 1.125917 |

| |
|---|
| 5 |
| 4 |
| 3 |
| 2 |
| 1 |
| 0.8 |
| 0.6 |
| 0.5 |
| 0.4 |
| 0.2 |
| 0 |

**Figure S4: Gene expression profile with exact numbers.**

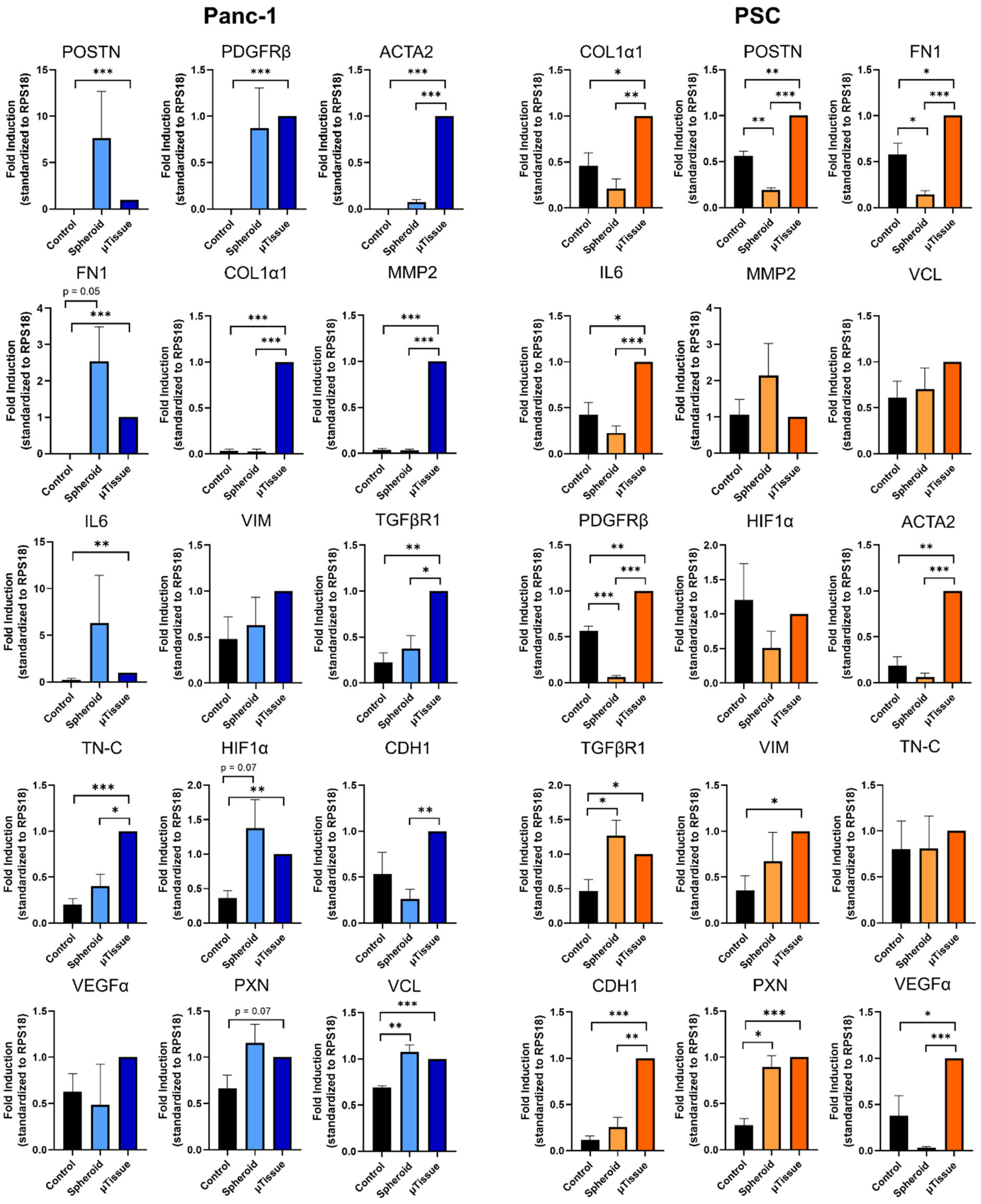


**Figure S5: Gene expression comparing 3D single culture of Panc-1, Panc-1/ PSC heterospheroids and Panc-1/ PSC µtissues.** Standardized to CK19 (left, blue graphs) and comparing 3D single culture of PSC, Panc-1/ PSC heterospheroids and Panc-1/ PSC µtissues standardized to FAP (right, orange graphs). Statistical analysis was performed by two-tailed Student's *t*-test. *$p < 0.05$, **$p < 0.01$, ***$p < 0.001$.

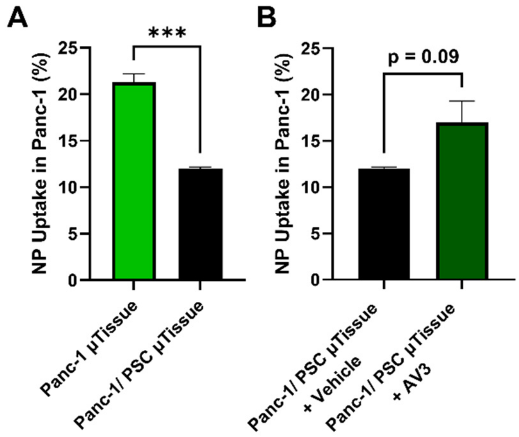


**Figure S6: Nanoparticle Uptake in Panc-1 cell core.** (A) Nanoparticle uptake in Panc-1 for Panc-1 µtissue versus Panc-1/ PSC µtissues. (B) Nanoparticle uptake in Panc-1 for Panc-1/ PSC µtissue treated with vechicle (DMSO) or 50 µM AV3. Statistical analysis was performed by two-tailed Student's *t*-test. ***$p < 0.001$.
